# Increased neutrophil apoptosis in chronically SIV-infected macaques

**DOI:** 10.1186/1742-4690-6-29

**Published:** 2009-03-24

**Authors:** Carole Elbim, Valérie Monceaux, Stéphanie François, Bruno Hurtrel, Marie-Anne Gougerot-Pocidalo, Jérome Estaquier

**Affiliations:** 1 Centre de Recherche des Cordeliers, Université Pierre et Marie Curie – Paris 6, UMR S 872, Paris, F-75006 France; Université Paris Descartes, UMR S 872, Paris, F-75006, France; 2 Institut Pasteur, Unité de Physiopathologie des Infections Lentivirales, Paris, F-75015, France; 3 AP-HP, Centre Hospitalier Universitaire Xavier Bichat, Service d'Immunologie et d'Hématologie, Paris, F-75018, France; 4 INSERM, U841, Faculté de Médecine de Créteil, Créteil, F-94010, France; 5 AP-HP, Hôpital Henri Mondor, Créteil, F-94010, France

## Abstract

Polymorphonuclear neutrophils (PMN) from chronically HIV-infected individuals have been reported to be more prone to die. However, although non-human primates models have been extensively used for improving our knowledge on T cell immunity, the impact of SIV-infection on PMN, in relationships with disease severity, has never been assessed. In our study, we demonstrate that PMN from Rhesus macaques (RMs) of Chinese origin chronically infected with the virulent strain SIVmac251 display increased susceptibility to undergo apoptosis as compared to PMN from RMs infected with the non-pathogenic SIVΔ*nef *strain. PMN apoptosis was significantly increased in RMs progressing faster to AIDS as compared to non-progressors RMs. Furthermore, the percentage of apoptotic cells correlated with PMN activation state reflected by increased CD11b expression and reactive oxygen species production. Interestingly, whereas inflammatory cytokines IL-8 and IL-1β prevent *in vitro *PMN death, the levels of those cytokines were low in RMs progressing towards AIDS. Altogether, increased PMN death during SIV infection is a new pathogenic effect associated with AIDS progression, adding to the long list of markers associated with disruption of defense against infection.

## Findings

Polymorphonuclear neutrophils (PMN) are key components of the first line of defense against pathogens [1]. PMN are terminally differentiated cells with a short life span; they die spontaneously by apoptosis and are then recognized and phagocytosed by macrophages [2]. Shortened PMN survival due to apoptosis may explain susceptibility to severe and recurrent infections in some pathological situations [3,4].

It has been reported that PMN functions are impaired in the latter stages of HIV infection[5]; increased PMN apoptosis has also been observed in HIV-infected patients having less than 200 CD4^+ ^cells/mm^3 ^[6-11]; however, the introduction of HAART has reduced spontaneous PMN apoptosis. Several lines of evidence suggest a key role of PMN, at least through defensin expression, in controlling viruses other than HIV or SIV [12-14]. In addition, human neutrophil α-defensins 1–4 have been reported to inhibit HIV-1 replication *in vitro *[15-17], and activated neutrophils have been demonstrated to exert cytotoxic activity against HIV-infected cells[18].

The use of non-human primate models, particularly SIV-infected Rhesus macaques (RMs), has allowed the detailed and sequential investigation of the events of SIV infection in terms of virus dynamics, immune response, and changes in the pool of CD4^+ ^cells [19]. Once the set-point phase is reached, the level of viral load predicts the rate of progression to AIDS [20-22]. Thus, SIVmac infection of RMs has proved to be an invaluable animal model for studies of AIDS pathogenesis, therapeutics, and vaccines. In particular, we and others have demonstrated that RMs of Chinese origin is a particularly relevant model to study human diseases [23-28]. Paradoxically, no studies have investigated, in SIV-infected RMs, possible PMN dysregulation, especially the propensity of PMNs to die, in relationships with disease severity.

Rhesus macaques (*Macaca mulatta*, RMs), of Chinese origin background, were confirmed, prior to infection, as seronegative for STLV-1 (Simian T Leukemia Virus type-1), SRV-1 (type D retrovirus), herpes-B viruses, and SIVmac. All animals were housed in compliance with French regulations for animal care and usage , and were inoculated intravenously with either pathogenic SIVmac251 strain [ten 50% animal infectious doses (AID)] or the live attenuated SIVmac251Δ*nef *strain [two hundred 50% AID].

All the animals were challenged with the same batch of virus, titrated *in vivo *in rhesus macaques, and were followed post-infection and studied 8 months later. RNA was extracted from plasma of SIV-infected monkeys, using the TRI REAGENT BD Kit (Molecular Research Center Inc., Cincinnati, Ohio). Real-time quantitative reverse transcriptase-polymerase chain reaction (RT-PCR) was used to determine viral loads as previously described [22]. Among SIV^+ ^macaques, slow progressors (n = 6) and moderate progressors (n = 5) were defined according to plasma viral load (<10^3 ^copies/ml and 10^3^–10^5 ^copies/ml, respectively), that predicts further disease progression in RMs of Chinese origin [20,21]. In contrast, in SIVΔ*nef*-infected monkeys, plasma viral load was always lower than 1.5 × 10^2 ^copies/ml. Moreover, as shown in Table 1, CD4^+ ^T cell counts were significantly decreased in moderate progressors as compared to SIV^+ ^slow progressors, SIVΔ*nef *or healthy (SIV^-^) macaques consistent with previous reports [20,21].

**Table 1:** Characteristics of the study population ^a^

	SIV^-^	SIVΔ*nef*	SIV^+ ^slow progressors	SIV^+ ^moderate progressors
	(n = 6)	(n = 4)	(n = 6)	(n = 5)
PMN count ^b^	1840 ± 259	1693 ± 325	1956 ± 252	1685 ± 245
Lymphocyte count ^b^	3104 ± 212	2970 ± 451	3317 ± 608	2354 ± 230
CD4^+ ^count ^b^	1127 ± 232	1086 ± 320	848 ± 149	317 ± 79 ^d, e^
CD8^+ ^count ^b^	651 ± 161	778 ± 184	1077 ± 277	1050 ± 232
Viral load ^c^	0	1.22 ± 0.42	38 ± 19 ^d, e^	1706 ± 699 ^d, e, f^

^a ^Monkeys were infected intravenously with 10 AID_50 _of the non-pathogenic nef-deleted SIVmac251 isolate (SIVΔ*nef*) or the pathogenic strain SIVmac251 (SIV^+^) and were studied 8 months later.

Values are means ± SEM.

^b ^PMN, lymphocytes, CD4^+ ^and CD8^+ ^counts are expressed per microliter.

^c ^Viral load is expressed as 10^2 ^copies per milliliter.

^d ^Significantly different from healthy controls (SIV^-^) (p < 0.05).

^e ^Significantly different from SIVΔ*nef *(p < 0.05).

^f ^Significantly different from SIV^+ ^slow progressors (p < 0.05).

**Figure 1:**
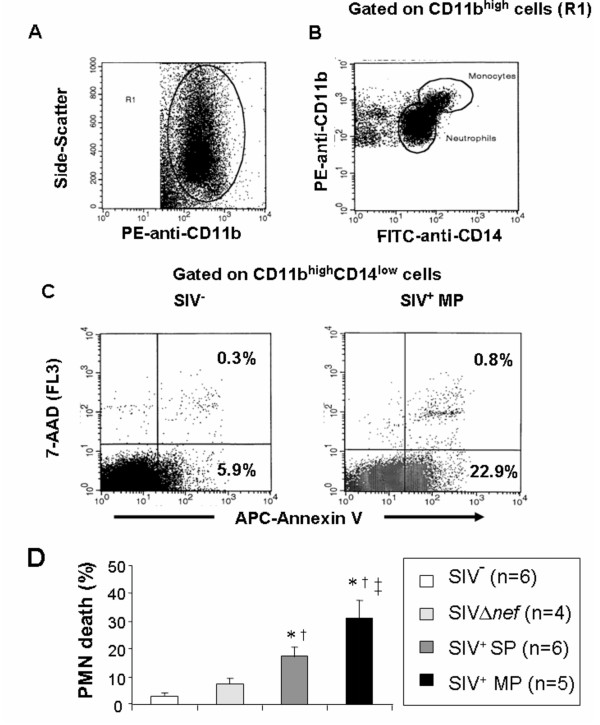
**PMN apoptosis during chronic infection of rhesus macaques with the pathogenic SIVmac251 strain**. **Panels A, B, and C **show the gating strategy to quantify apoptosis. A) Dot plot showing anti-CD11b-PE staining against the side-scatter parameter. A first gate (R1) was drawn around CD11b^+ ^cells. B. Dot plot showing anti-CD11b-PE and anti-CD14-FITC staining, gated on R1. A second gate (R2) was drawn on CD14^low ^cells in order to eliminate monocytes (CD14^high ^cells) from the analysis. C) The combination of annexin V-APC and 7-AAD staining distinguished early apoptotic cells (annexin V^+^, 7-AAD^-^) and late apoptotic cells (annexin V^+^, 7-AAD^+^) in an SIV^- ^macaque and in an SIV^+ ^macaque moderate progressor (SIV^+ ^MP) (day 240) after incubating whole blood at 37°C for 4 hours (T4h). **Panel D**. Eleven SIV^+ ^(six slow progressors, SIV^+ ^SP and five moderate progressors, SIV^+ ^MP) and four SIVΔ*nef *macaques were studied after 8 months of infection. Apoptosis was studied after incubating whole blood at 37°C for 4 hours (T4h). Results are expressed as percentage of annexin V^+^/7-AAD^- ^cells (early apoptotic cells). Data are reported as means ± SEM. Comparisons were based on ANOVA and Tukey's posthoc test, using Prism 3.0 software. * Significantly different from healthy controls (SIV^- ^group) (p < 0.05); ^† ^Significantly different from SIVΔ*nef *macaques (p < 0.05); ^†† ^Significantly different from SIV^+ ^SP (p < 0.05).

To quantify PMN apoptosis, samples were analyzed directly on the whole blood cooled to 4°C to avoid non-specific activation due to isolation procedure [29,30]. PMN apoptosis was measured after 4 hours of incubation in 24-well tissue culture-plates at 37°C with 5% CO_2_. Apoptosis was quantified by allophycocyanin (APC)-conjugated annexin V and 7-amino-actinomycin D (7-AAD) staining as previously described [29,30]. Whole blood samples (100 μl) were washed twice in PBS, incubated with FITC-anti-CD14 and PE-anti-CD11b monoclonal antibodies (mAbs) for 15 minutes, and then incubated with APC-annexin V for 15 minutes. After dilution in PBS (500 μl) the samples were incubated with 7-AAD at room temperature for 15 minutes and analyzed immediately by flow cytometry. PMN were identified as CD11b^+^CD14^low ^cells (Figure 1A and 1B).

After 4 hours of incubation at 37°C, PMN apoptosis was significantly increased in SIV^+ ^macaques relative to SIVΔ*nef *and healthy controls (Figure 1D). The fact that SIVΔ*nef *macaques showed reduced susceptibility to apoptosis as compared to SIV^+ ^animals is in keeping with a previous report that the apathogenic strain is associated with milder immune dysfunction and has a lower plasma viral load [31]. Moreover, PMN apoptosis was significantly increased in moderate progressors as compared to slow progressors (percentage of annexin V^+ ^cells: 33.0 ± 5.1 and 15.7 ± 1.5, in moderate and slow progressors respectively). In addition, PMN apoptosis in individual SIV^+ ^macaques correlated negatively with the corresponding CD4^+ ^T cell counts and positively with plasma viral load (ρ = -0.59, p = 0.05 and ρ = 0.91, p = 0.0003, respectively) (correlation was identified by means of the Spearman rank correlation coefficient ρ). These results suggest that the rate of PMN apoptosisis directly related to the speed of disease progression. In accordance with the general view that many cell types undergo death in a caspase-independent manner [32,33], preincubation of whole blood samples with the broad caspase inhibitor CBz-Val-Ala-DL-Asp(Ome)-fluoromethylketone (z-VAD-fmk) (10 μM) for 15 minutes did not prevent PMN death in SIV^+ ^macaques (data not shown), whereas the same compound prevented Fas-mediated apoptosis of CD8^+ ^T cells in agreement with previous reports [30,32,34]. Altogether, these results suggest that PMN are abnormally primed to undergo death through a caspase-independent pathway in SIV-infected macaques progressing more rapidly to AIDS.

Whereas we found an increased PMN propensity to die, this was not reflected by an apparent decline of PMN counts (Table 1). This result contrasts with our recent data observed during the acute phase of SIV infection in Chinese RM demonstrating that PMN death is associated with neutropenia early after infection [30]. Increased emigration from the bone marrow of mature PMN could be an explanation compensating for the absence of apparent depletion chronically infected macaques. Therefore, we decided to analyze the basal activation status of PMN in the periphery, by measuring CD11b expression and ROS production. Superoxide anion O_2_^- ^production was measured with a flow cytometric assay derived from the hydroethidine (HE) oxidation technique [29,30].

**Figure 2:**
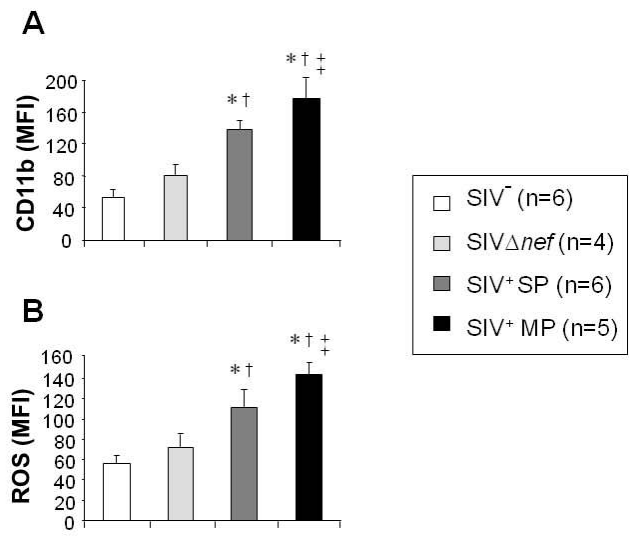
**PMN functions during chronic infection of rhesus macaques with the pathogenic SIVmac251 strain or the attenuated SIVΔ*nef *strain**. Basal CD11b expression on the PMN surface (A) and basal ROS production (B) were studied in whole blood samples. Results are expressed as Mean Fluorescence Intensity (MFI). Data are reported as means ± SEM. Comparisons were based on ANOVA and Tukey's posthoc test, using Prism 3.0 software. * Significantly different from healthy controls (SIV^- ^group) (p < 0.05); ^† ^Significantly different from SIVΔ*nef *macaques (p < 0.05); ^†† ^Significantly different from SIV^+ ^SP (p < 0.05).

Both CD11b expression (Figure 2A) and ROS production (Figure 2B) of resting PMN (maintained at 4°C) were significantly increased in both slow and moderate progressors relative to healthy and SIVΔ*nef *controls. Furthermore, PMN from moderate progressors exhibited increased CD11b expression and ROS production as compared to slow progressors. Interestingly, the percentage of apoptotic cells correlated with basal PMN activation status (ρ = 0.69, p = 0.01 and ρ = 0.71, p = 0.01, for CD11b expression and ROS production, respectively). The consequences of PMN activation generating general oxidative stress molecules might include an increased PMN susceptibility to apoptotic death during the chronic phase of infection [35]. In addition, these results support the idea that increased granulopoiesis in bone marrow leads to a compensatory release of mature PMN. Interestingly, PMN activation has been reported in the bone marrow of chronically SIV-infected macaques [36], contrasting with a defect in bone marrow lymphopoiesis [37,38]. Infact, reciprocal dynamics of the bone marrow lymphocyte and neutrophil populations lead to cellular competition within a developmental niche. In particular, blocking bone marrow lymphopoiesis results in the specific and reciprocal expansion of the granulocytic compartment in bone marrow [39].

Our results showed that, at 8 months post-inoculation (p.i.), the extent of PMN apoptosis is higher than that observed at 2 months p.i., while the levels of viral replication remain quite similar [30]. In addition, during the acute phase, the levels of 10^7 ^copies/ml in RMs infected with the pathogenic strain is associated with PMN death; interestingly in RMs infected with the live attenuated Δ*nef *strain, despite a viral load of 10^5 ^copies/ml at the peak (day 14 p.i.), no PMN death was observed [30]. This level of viral replication corresponds to that observed during the chronic phase. Altogether, these data suggest that, while a certain threshold of viral particles is required for a direct effect on cell death, extracellular factors could participate in PMN dysregulation. Because it has been shown that inflammatory cytokines inhibit PMN apoptosis [40,41], we then determined in the plasma the amount of IL-8, TNF-α, and IL-1β. Blood was collected in sterile EDTA-treated vacuum tubes and immediately centrifuged at 400 *g *for 15 minutes at 4°C. IL-8, TNF-α, and IL-1β were detected simultaneously by using the human inflammatory cytokine cytometric bead array (CBA) kit (BD Pharmingen), which has been validated for cytokine measurements in RMs after Toll-like receptor (TLR) stimulation (data not shown).

**Figure 3:**
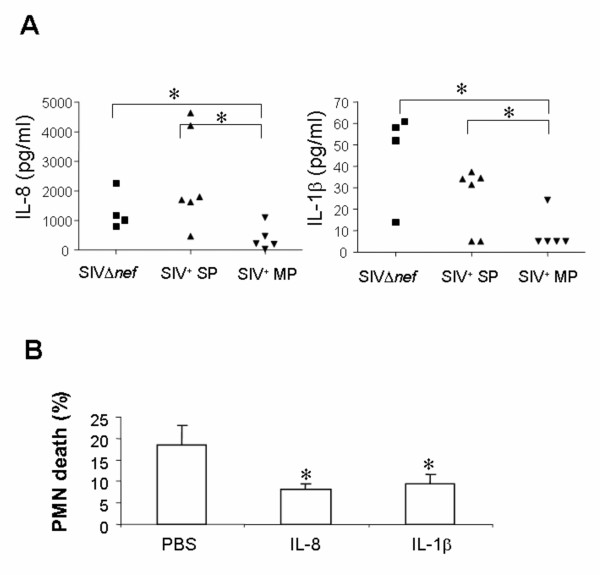
**Levels of pro-inflammatory cytokines during chronic infection of rhesus macaques with the pathogenic SIVmac251 strain**. A) IL-8 and IL-1β levels during chronic infection of rhesus macaques with the pathogenic SIVmac251 strain (slow progressors SIV^+ ^SP, and moderate progressors, SIV^+ ^MP) or the attenuated SIVΔ*nef *strain. Plasma levels of IL-8 and IL-1β were measured by using the inflammatory cytokine cytometric bead array (CBA).* Significantly different (p < 0.05). B) Effect of IL-8 and IL-1β on PMN survival. Whole blood samples from SIV^+ ^rhesus macaques were incubated at 37°C for 4 hours either with IL-8 (100 ng/ml), IL-1 (100 ng/ml) or PBS. Results are expressed as percentage of annexin V^+^/7-AAD^- ^cells (early apoptotic cells). Data are reported as means ± SEM. Comparisons were based on ANOVA and Tukey's posthoc test, using Prism 3.0 software. * Significantly different from samples incubated with PBS alone (p < 0.05).

Firstly, we found that IL-8 and IL-1β plasma levels were significantly lower in SIV^+ ^moderate progressors as compared to SIV^+ ^slow progressors and SIVΔ*nef *macaques (Figure 3A). Secondly, pre-incubation of whole blood samples from moderate progressors with IL-8 (100 ng/ml) or IL-1β (100 ng/ml) for 4 hours significantly reduced PMN apoptosis as compared to samples incubated with PBS [percentage of annexin V^+ ^cells: 8.2 ± 1.3, 9.5 ± 2.1 and 18.5 ± 4.3 for the samples incubated with IL-8, IL-1β (R&D), or PBS, respectively] (Figure 3B). In contrast, TNF-α was undetectable in the plasma of SIV-infected macaques. The absence of detection of TNF-α in chronically SIV-infected rhesus macaques is consistent with a recent report [42]. Indeed, the authors did not observe increased levels of pro-inflammatory cytokines despite increased levels of plasma LPS. Thus, the absence of inflammatory cytokines might lead to an abnormal tendency of PMN to die.

Finally, one consequence of such abnormal PMN apoptosis could be to facilitate the dissemination of SIV/HIV *in vivo *by modulating immune responses. Apoptotic cells are sources of biologically active oxidized phospholipids which serve as recognition signals on apoptotic cells, facilitating phagocytosis by macrophages [43]. Engulfment of apoptotic PMN has been shown to inhibit the production of pro-inflammatory mediators by macrophages, through the secretion of anti-inflammatory cytokines such as TGF-β [44]. In this context, we recently demonstrated that TGF-β is increased in the tissues of SIV-infected RMs [25]. Such anti-inflammatory events can inhibit antigen presentation and promote microbial growth within macrophages [45], HIV replication [46], as well as the expansion of IL-17-producing cells [47].

In conclusion, our data demonstrate for the first time, to our knowledge, in SIV-infected macaques abnormal PMN deaths that increased in monkeys progressing faster to AIDS. This abnormality might therefore participate in the general immune defect leading to clinical outcomes in SIV infection.

## Competing interests

The authors declare that they have no competing interests.

## Authors' contributions

CE, BH, MAGP and JE designed the study. CE and SF performed neutrophil studies and the statistical analysis; VM performed viral load measurements. VM participated in the design of the study and performed viral load measurements. CE and JE wrote the manuscript. All authors read and approved the manuscript.
